# Supplementary material for: Nanoparticle-Based Therapeutic Strategies for Enhanced Pancreatic Ductal Adenocarcinoma Immunotherapy
Source: Pharmaceutics. 2022 Sep 24;14(10):2033. doi: 10.3390/pharmaceutics14102033 (PMC9607590; doi:10.3390/pharmaceutics14102033)
Supplement: Supplementary file 1 [file pharmaceutics-14-02033-s001.zip › pharmaceutics-1817252-supplementary.pdf]

Supplement Table S1. Published clinical trials of other immunotherapy in PDAC.

	Therapeutic strategy	Phase	Stage	Number of Patients	Objective Response Rate	Median PFS(months)	Median OS(months)	immune-related adverse events (≥ grade 3)	Publication year	NCT number	Ref
Cancer vaccine	mutant ras peptides+GM-CSF	I/II	PDAC	48	NA	NA	NA	NA	2001	NA	PMID: 11291084
	GEM+TG01/GM-CSF	I/II	resected RAS-mutant PDAC	32	NA	NA	34.3	NA	2020	NCT02261714	PMID: 32063605
	GVAX+ipilimumab vs FOLFIRINOX	II	mPDAC	40 vs 42	NA	2.4 vs 5.55	9.8 vs 14.7	36% (ipi 3mg/kg) vs 44% (ipi 10mg/kg)	2020	NCT01896869	PMID: 32591464
	GVAX	II	resectable PDAC	60	NA	17.3	24.8	NA	2011	NCT00084383	PMID: 21217520
	Cy(cyclophosphamide)/GVAX + CRS-207 + nivolumab vs Cy(cyclophosphamide)/GVAX + CRS-207	II	mPDAC	51 vs 42	4% vs 2%	NA	5.9 vs 6.1	35.3%vs11.9%	2020	NCT02243371	PMID: 29997287

	Cy/GVAX followed by four doses of CRS-207<br>vs<br>Cy/GVAX followed by six doses of Cy/GVAX	II	mPDAC	62 vs 29	NA	NA	6.1 vs 3.9	NA	2015	NCT01417000	PMID: 25584002
	GVAX<br>vs<br>GVAX+cyclophosphamide at 200 mg/m2 one day before each vaccination<br>vs<br>GVAX+cyclophosphamide at 100 mg once daily for one week on and one week off starting on the day of vaccination	II	resectable PDAC	22 vs 23 vs 21	NA	NA	34.2vs15.4vs16.5	NA	2021	NCT00727441	PMID: 33277370
	GVAX+CRS-207(live, attenuated Listeria monocytogenes expressing mesothelin)<br>vs<br>CRS-207<br>vs<br>single-agent chemotherapy	IIb	mPDAC	68 vs 58 vs 43	SD +PR: 23.5% vs 13.8% vs 11.6%	2.3 vs 2.1 vs 2.1	3.7 vs 5.4 vs 4.6	46.8%vs36.8%vs27.8%	2019	NCT02004262	PMID: 31126960
	Nab-P+GEM+IL-2+GM-CSF	II	advanced PDAC	64	43.75%	5.7	14.2	59.38%	2021	NCT03768687	PMID: 34722242

	ipilimumab vs ipilimumab+GVAX	Ib	previously treated PDAC	15 vs 15	3 SD vs 2 SD	NA	3.6 vs 5.7	20% vs 20%	2014	NCT00836407	PMID: 23924790
	chemotherapy vs chemotherapy+sequential GV1001 vs chemotherapy+concurrent GV1001	III	locally advanced or metastatic PDAC	358 vs 350 vs 354	NA	NA	7.9 vs 6.9 vs 8.4	NA	2014	ISRCTN4382138	PMID: 24954781
	KIF20A-66 peptide	I	advanced pancreatic cancer	29	DCR 72%(SD)	56 days	142days	NA	2013	UMIN000004919	PMID: 24237633
	DC vaccines	I	metastatic or unresectable PDAC	12	NA	3	7.7	NA	2017	NCT01410968	PMID: 28388966
	DC-CIK+S-1 vs DC-CIK vs chemotherapy vs supportive care	A Prospective Study	advanced pancreatic cancer	25 vs 11 vs 4 vs 7	NA	136 days vs 85 days vs 92 days vs 43 days	212 days vs 128days vs 141days vs 52days	0	2017	NCT01781520	PMID: 28611200

	DC vaccine+GEM+/S-1 vs DC vaccine+lymphokine-activated killer cell therapy+GEM+/S-1	retrospective study	advanced pancreatic carcinoma	15 vs 34	0%(CR) vs 5.9%(CR); 13.3%(PR) vs 8.8%(PR); 6.7%(SD) vs 26.5%(SD)	NA	NA	0	2012	NA	PMID: 21792083
	chemoradiotherapy+algenpantucel-L	II	resected pancreatic cancer	70	NA	NA	NA	＜12%	2013	NCT00569387	PMID: 23229886
	neoadjuvant chemotherapy+chemoradiation vs neoadjuvant chemotherapy+algenpantucel-L	III	Borderline Resectable or Locally Advanced Unresectable Pancreatic Cancer	158 vs 145	NA	13.4 vs 12.4	14.9 vs 14.3	75% vs 81%	2022	NCT01836432	PMID: 33630475
	K-ras vaccine	I/II	resected PDAC	23	NA	NA	27.5	0%	2010	NA	PMID: 20473937
	WT1 and/or MUC1peptide-loaded DC vaccination+chemotherapy	I/II	advanced pancreatic cancer	48	14.6%	8.1	15.1	0%	2021	NA	PMID: 34919493
	TG01/GM-CSF+GEM	I/II	patients with resected RAS-mutant adenocarcinoma	32	94% immune response	NA	33.3	NA	2020	NCT02261714	PMID: 32063605

			of the pancreas								
**CART**	mesothelin-specific CART	I	chemotherapy-refractory metastatic PDAC	6	Disease stabilized in 2 patients, with progression-free survival times of 3.8 and 5.4 months	NA	NA	no dose limiting toxicities	2018	NA	PMID: 29567081
	CAR T-EGFR	I	metastatic pancreatic carcinoma	16	Of 14 evaluable patients, four achieved partial response for 2-4 months, and eight had stable disease for 2-4 months	3.0	4.9	NA	2020	NCT01869166	PMID: 32527643
**Oncolytic virus**	pembrolizumab+oncolytic virus pelareorep+chemotherapy	Ib	advanced pancreatic adenocarcinoma	11	9%	2.0	3.1	9.1%	2020	NA	PMID: 31694832
**ACT**	MUC1-DCs+MUC1-CTLs+GEM	NA	unresectable or recurrent pancreatic cancer	42	CR:2.4%;PR:7.1%; SD:52.4%	NA	13.9	NA	2014	NA	PMID: 24947606
	MUC1-DC+cytotoxic T lymphocyte (CTL)	NA	unresectable or recurrent pancreatic	20	1 patient CR; 5 patients SD	NA	9.8	0%	2008	NA	PMID: 18383873

			cancer								
	GEM+autologous γδ T-cell transfer vs GEM	I	curatively resected pancreatic cancer	28 vs 20	NA	NA	NA	NA	2017	University Hospital Medical Information Clinical Trials Registry identifier 000000931	PMID: 28188072
**NK cell**	irreversible electroporation (IRE) vs IRE-NK	NA	unresectable (stage III/IV) pancreatic cancer	16 vs 19	Stage III:50.0% vs 63.2%; Stage IV:21.4% vs 26.3%;	7.9 vs 9.1	12.2 vs 13.6	NA	2017	NA	PMID: 28871458
**CD40**	CP-870,893(anti-CD40)+GEM	I	advanced PDAC	22	19%, 4 PR	5.2	8.4	NA	2013	NA	PMID: 33497362
	APX005M(anti-CD40)+GEM+ Nab-P vs APX005M(anti-CD40)+GEM+ Nab-P+nivolumab	Ib	mPDAC	6 (0.1 mg/kg APX005M); 6 (0.3 mg/kg APX005M); vs 6 (0.1 mg/kg APX005M); 6 (0.3 mg/kg	67% (0.1 mg/kg APX005M); 33% (0.3 mg/kg APX005M); vs 67% (0.1 mg/kg APX005M); 67% (0.3 mg/kg	12.5 (0.1 mg/kg APX005M); 10.4 (0.3 mg/kg APX005M);	12.7 (0.1 mg/kg APX005M); 20.1 (0.3 mg/kg APX005M);	85% (0.1 mg/kg APX005M); 100% (0.3 mg/kg APX005M); vs 88% (0.1 mg/kg APX005M);	2021	NCT03214250	PMID: 33387490

				APX005M);	APX005M)	vs 10.8 (0.1 mg/kg APX005M); 12.4 (0.3 mg/kg APX005M)	vs 15.9 (0.1 mg/kg APX005M); not estimable (0.3 mg/kg APX005M)	100% (0.3 mg/kg APX005M)			
	sotigalimab(anti-CD40)+nivolumab	II	mPDAC	37(nivo/chemo)vs31(sotiga/chemo)vs 31(sotiga/nivo/chemo)	50% vs 33% vs 31%	6.4 vs 7.3 vs 6.7	16.7 vs 11.4 vs 10.1	NA	2022	NCT03214250	PMID: 35662283
**Others**	ibrutinib (BTK inhibitor)+durvalumab	Ib/II	relapsed/refractory PDAC	49	2%	1.7	4.2	78%	2019	NCT02401048	PMID: 31230047
	acalabrutinib (BTK inhibitor) vs acalabrutinib + pembrolizumab	II	advanced PDAC	37 vs 40	0%	1.4	NA	14.3% vs 15.8%	2020	NCT02362048	PMID: 32114502
	nivolumab + mogamulizumab (Anti-CCR4)	I	mPDAC	15	1/15	1.8	6.5	NA	2019	NCT02476123	PMID: 31455681
	PF-04136309（CCR2 inhibitor）+Nab-P+GEM	Ib	mPDAC	21	23.8%	NA	NA	NA	2020	NCT02732938	PMID: 31297636

	BL-8040 (CXCR4 inhibitor) + pembrolizumab + chemotherapy	IIa	mPDAC	22	0.27%	NR	NR	NA	2020	NCT02826486	PMID: 32451495
	irreversible electroporation+nivolumab	Ib	locally advanced PDAC	10	NA	6.3	18	70%	2020	NCT03080974	PMID: 32631655
	Neoadjuvant Oregovomab(CA125 monoclonal antibody)-Based Chemoimmunotherapy+SBRT	I/II	locally advanced PDAC	11	3CR;4SD	8.6	13	54.5%	2019	NA	PMID: 31513018

GM-CSF: Granulocyte macrophage colony stimulating factor; GVAX: GM-CSF cell-based vaccines; GEM: gemcitabine; Nab-P: nab-paclitaxel; LAPC: locally advanced pancreatic cancer; mPDAC: metastatic pancreatic ductal adenocarcinoma; SBRT: stereotactic body radiation therapy; NA:not available .
